# Clipping towards clarity: Significant role of simple nail clipping in the diagnosis of nail unit melanoma

**DOI:** 10.1016/j.jdcr.2026.05.016

**Published:** 2026-05-20

**Authors:** Michael Harouni, Kaviyon Sadrolashrafi, Narciss Mobini

**Affiliations:** a Applied Health Sciences at UNLV, University of Nevada at Las Vegas (UNLV), Las Vegas, Nevada; b Southern Hills Hospital and Medical Center, Las Vegas, Nevada; c Department of Internal Medicine, Kirk Kerkorian School of Medicine at UNLV, Las Vegas, Nevada; d Associated Pathologists Chartered in Affiliation with Quest Diagnostics, Las Vegas, Nevada

**Keywords:** melanocyte remnants, melanonychia, nail clipping, nail dystrophy, nail melanoma, subungual melanoma, ungual melanoma

## Introduction

Malignant melanoma of the nail unit is a rare, but potentially life-threatening disease that may go undiagnosed until its later stages due to its clinical resemblance to more common benign nail disorders.^1^^,^^2^ The most common clinical presentation is brown-black discoloration of the nail plate or longitudinal melanonychia. Although this presentation is more commonly seen with external trauma resulting in secondary subungual hematoma, it may also represent underlying pigmented onychomycosis, bacterial (pseudomonas) infection, pyogenic granuloma, medication-induced hyperpigmentation, a benign melanocytic process such as lentigo or nevus, or a malignant lesion such as pigmented squamous cell carcinoma in situ/Bowen’s disease.^2^^,^^3^ Delayed diagnosis often results in a poor prognosis; therefore, having a high index of suspicion is critical for early detection of ungual melanoma.^1^, ^2^, ^3^, ^4^

We report a case highlighting how a simple nail clipping ultimately led to the diagnosis of an invasive nail unit melanoma (NUM), which altered the patient’s care trajectory and underscores the potentially life-saving diagnostic value of this routine procedure.

## Case report

**Fig 1:**
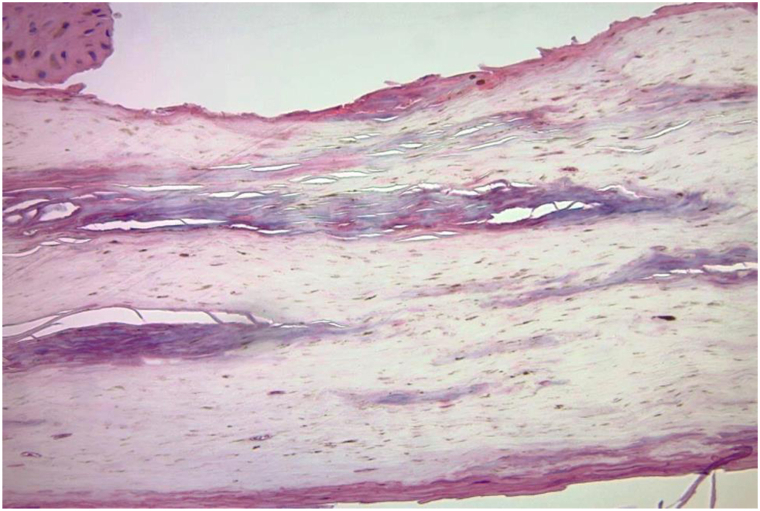
Dystrophic nail plate containing brown pigment (melanin), scattered melanocytes, and melanocyte remnants, nail clipping. H&E, magnification 100×.

**Fig 2:**
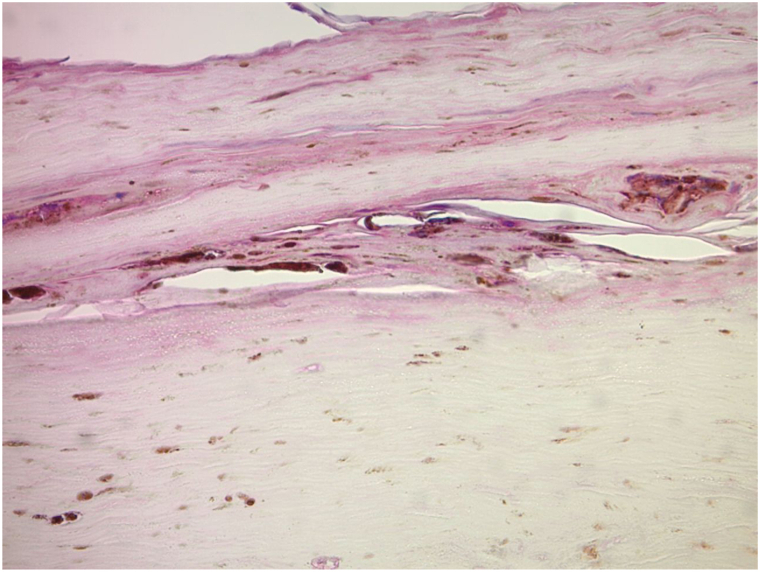
Dystrophic nail plate containing melanin, scattered melanocytes, and larger melanocyte remnants, nail clipping. H&E, magnification 200×.

**Fig 3:**
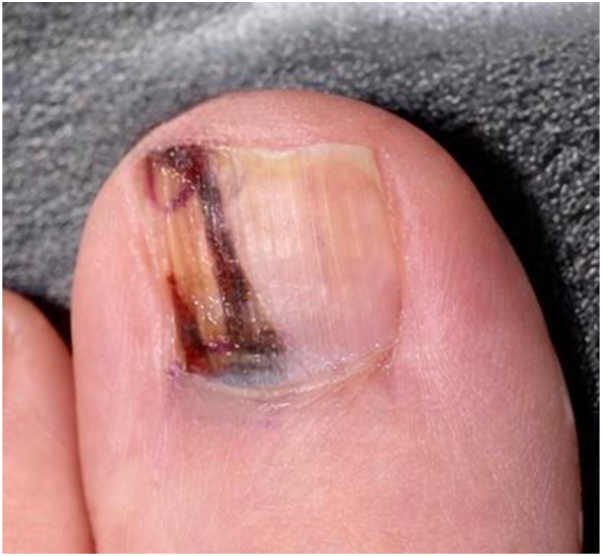
Irregular longitudinal melanonychia of left hallux (prior to nail matrix biopsy).

A 42-year-old woman presented for evaluation of discoloration and thickening of her left great toenail. These symptoms had been present for 2 years and were initially diagnosed as a subungual hematoma related to possible trauma, recalcitrant fungal infection, and nail dystrophy. During this time, the patient’s nail lesion had gradually increased in size and pigmentation. The patient received several courses of topical and systemic antifungal treatments, which failed to improve her condition. Eventually, a nail clipping was performed primarily to rule out chronic, nonresponsive onychomycosis or onychodystrophy. Histopathologic evaluation revealed dystrophic changes in the nail plate, subungual parakeratosis, and collections of serum crust with bacterial colonies. A Periodic Acid Schiff stain was negative for fungi. Of note, brown pigmentation was present within the thickened nail plate (Figs 1 and 2). This pigment was composed of melanin granules and pigmented melanocytes, a finding suggestive of an underlying melanocytic process. Based on this abnormal observation, a deep biopsy of the nail unit was recommended for further evaluation. The patient was referred to a dermatologic surgeon. Physical examination revealed longitudinal melanonychia involving the left hallux, approximately 6.0 mm in width (Fig 3).

**Fig 4:**
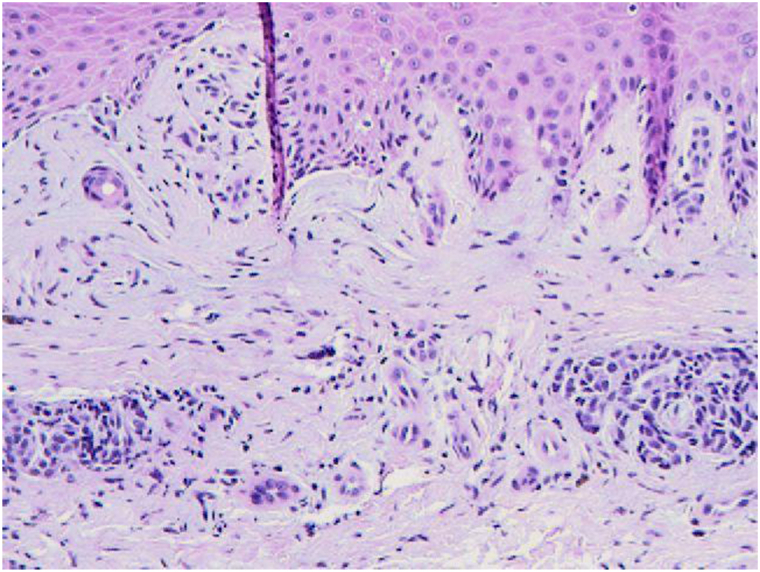
Melanoma in situ and invasive (Breslow thickness of 0.5 mm), nail matrix, Mohs excision. H&E, magnification 100×.

The nail matrix biopsy demonstrated malignant melanoma in situ. The patient subsequently underwent Mohs micrographic surgery for a complete removal of the lesion. Examination of the debulked specimen revealed extensive residual melanoma in situ with 0.5 mm of invasion into the underlying dermis (Fig 4). The final pathologic staging was pT1a. A thorough metastatic workup, including imaging and laboratory studies, showed no evidence of regional or distant spread. The patient remains under close dermatologic surveillance with no signs of recurrence to date.

## Discussion

NUM is an uncommon subtype of melanoma, accounting for approximately 1% to 2.5% of all cutaneous melanomas in White populations, 23% in Japanese populations, and 25% in Afro-Caribbean populations.^5^^,^^6^ It frequently presents as longitudinal melanonychia; however, up to 25% of cases may be amelanotic and mimic chronic paronychia, lichen planus, or vascular lesions, further complicating timely recognition.^2^^,^^5^^,^^7^ The nail matrix is the main site of melanocyte production in the nail unit. Since melanocytes do not normally reach the keratogenous zone in benign proliferations, the presence of melanocytes or their remnants in the keratinous nail plate is a very useful clue to the diagnosis of melanoma, particularly in adults.^1^^,^^8^ This finding is akin to pagetoid spread seen in the skin. Several reports have emphasized the importance of obtaining adequate matrix sampling when evaluating suspicious melanonychia.^1^, ^2^, ^3^ Clinical warning features such as band width greater than 3 mm, progressive widening, irregular pigmentation, periungual extension (Hutchinson sign), or involvement of a single digit in adulthood should prompt evaluation.^5^ Dermoscopy alone is insufficient to exclude malignancy, and histopathologic confirmation should remain as the gold standard.^1^^,^^5^

Many clinicians are reluctant to perform nail matrix biopsy or are unfamiliar with the procedure, and it is also not preferred by patients. These factors may lead to easily avoidable and potentially dangerous delays in accurate diagnoses. Although typically used to evaluate onychomycosis or onychodystrophy in daily practice, nail clipping can potentially help reveal critical clues in the diagnosis of malignant melanoma. Dermatopathologists should be on the lookout for pigmentation in nail plates, which may be identified as small or large melanocyte remnants, atypical pigmented material, or melanocytes. Melanocytic immunomarkers can also be used as part of routine histopathology to characterize melanocytes or their remnants better, although results vary across studies.^1^^,^^2^^,^^9^^,^^10^ Our case was positive for Sox10 and Panmelanoma (not shown). Detection of melanin in the nail clipping samples should prompt further investigation and nail matrix biopsy as the immediate next diagnostic step.

As features of NUM can be subtle and deceiving both clinically and histopathologically, clear communication between the clinician and the dermatopathologist is critically important. Providing relevant clinical information beyond the desire to rule out fungal infection or nail dystrophy will most likely help in an in-depth evaluation. This information may include the chronicity or persistence of nail changes, atypical or progressive pigmentation patterns, and prior treatment failures. Awareness of unusual types of nail dystrophies in adults, particularly when alarming features such as a monodactylous involvement are present, is essential to form a comprehensive differential diagnosis.

Our case highlights the critical role of nail clippings as an early detection/diagnostic tool in NUM. Clinicians should maintain a high index of suspicion for any monodactylous nail changes in adults. This simple, quick, and tolerable procedure may facilitate earlier detection of malignant melanoma, minimize the risk of progression, and improve patient outcomes.

## Conflicts of interest

None disclosed.
